# Development of near-zero water consumption cement materials via the geopolymerization of tektites and its implication for lunar construction

**DOI:** 10.1038/srep29659

**Published:** 2016-07-13

**Authors:** Kai-tuo Wang, Qing Tang, Xue-min Cui, Yan He, Le-ping Liu

**Affiliations:** 1 School of Chemistry and Chemical Engineering and Guangxi Key Lab of Petrochemical Resource Processing and Process Intensification Technology, Guangxi University, Nanning, 530004, China

## Abstract

The environment on the lunar surface poses some difficult challenges to building long-term lunar bases; therefore, scientists and engineers have proposed the creation of habitats using lunar building materials. These materials must meet the following conditions: be resistant to severe lunar temperature cycles, be stable in a vacuum environment, have minimal water requirements, and be sourced from local Moon materials. Therefore, the preparation of lunar building materials that use lunar resources is preferred. Here, we present a potential lunar cement material that was fabricated using tektite powder and a sodium hydroxide activator and is based on geopolymer technology. Geopolymer materials have the following properties: approximately zero water consumption, resistance to high- and low-temperature cycling, vacuum stability and good mechanical properties. Although the tektite powder is not equivalent to lunar soil, we speculate that the alkali activated activity of lunar soil will be higher than that of tektite because of its low Si/Al composition ratio. This assumption is based on the tektite geopolymerization research and associated references. In summary, this study provides a feasible approach for developing lunar cement materials using a possible water recycling system based on geopolymer technology.

The NASA Human Exploration Destination Systems Roadmap mentions the development of technologies for shielding astronauts from radiation during missions to the lunar surface and Mars between 2017 and 2022 (1). The Chinese lunar exploration project is listed as one of 16 major projects in the 2006–2020 “National long-term science and technology development plan”, which actualizes the project of manned landing on the Moon and establishing a lunar base by 2030^1^. However, the lunar surface environment poses difficult challenges for building long-term lunar bases on the Moon^2^. Scientists and engineers propose the creation of habitats using lunar building materials. These lunar building materials will be designed and prepared based on the following primary considerations^3^: [1] resistance to severe lunar temperature cycles (102.4 K to 387.1 K), [2] stability in a vacuum environment, [3] minimal water requirements, and [4] sourced from local Moon materials. The development of permanent lunar bases is constrained by the performance of construction materials and the transportation cost from Earth. Thus, the preparation of lunar building materials that use lunar resources is preferred^3^.

A structural analysis and a preliminary design of a concrete lunar base was first reported by Beyer^4^. Because the preparation of cement concrete needs water, the project is difficult to realize in the lunar environment. To avoid the difficulties of mixing concrete on the Moon owing to a lack of water, it has been suggested that a sulfur concrete should be studied^5^. However, the strength, durability and refractoriness of sulfur concrete are worse than those of cement concrete. Thus, cement concrete is a preferable construction material for the lunar environment. However, water is one of its major components, and it is a scarce resource on the Moon. In recent years, Saal^6^ and Hauri^7^ demonstrated that there may be water on the Moon. However, the Lunar Prospector Mission team indicated that the moisture content in the regolith at the bottom of the crater might be between 0.3% and 1%^8^. Therefore, the utilization of such a low level of water is not realistic, and the cost is high. Thus, a type of lunar cement material that does not use or uses very little water is preferred when building permanent structures on the Moon.

In general, cement materials develop hydrates and attain their bonding strength after mixing with a quantity of water that is approximately 20% to 30% of the cement mass^9^. While studying geopolymer materials, researchers found that these materials need only a small amount of water during the initial reaction stage, and most of the water can then be removed without affecting the strength^10^. If the water is collected and reused, its consumption will be significantly less than the consumption in traditional cement materials.

Geopolymers were first introduced by Davidovits in 1978^10^. Geopolymers are amorphous or quasi-crystalline inorganic polymer gels with three-dimensional network structures. These materials are composed of hydrated structures and are prepared from natural minerals or solid wastes via the polymerization of SiO_4_ and AlO_4_ tetrahedra. Compared with traditional cement materials, the molecular structure of geopolymers is formed via the dehydration polycondensation process as opposed to combining with water to form a molecular structure that does not rely on the presence of water^10^. Furthermore, geopolymers are materials that shield radioactivity^11^. A representative geopolymer system is fabricated from alkaline- or alkali-silicate-activated metakaolin with a wide compositional range, which is represented as [Na(K)]_2_O-mAl_2_O_3_-nSiO_2_ with m ≈1 and 2 ≤ n ≤ 6^12^. Most studies have suggested that the properties of geopolymers are restricted by the Si/Al compositional range. According to previous studies^13^,^14^, the Na(K)-Al-Si or Ca(Mg)-Al-Si gels form within a narrow Si/Al compositional range. Furthermore, the alkali activation activity increases with a decrease in the Si/Al ratio within a narrow range.

Tektites are natural glass objects of unknown origin. They have been found on Earth and have been identified as Australian tektites, Southeast Asian tektites, American glass meteorites and tektites from Chinese Hainan and Leizhou Peninsula^15^. Lei Gong Mo is a type of black tektite. It is found in southern China. Its composition is shown in Table 1. Based on the composition of several mineral materials listed in Table 1, the molar ratio of Si/Al was calculated to be 1.13, 1.41, 2.32, 2.64 and 4.88. In addition, lunar soil is composed of various types of particles including rock fragments, mono-mineralic fragments, and various types of glasses, including agglutinate particles and volcanic and impact spherules^16^,^17^,^18^,^19^. The compositions of different lunar soil samples are given in Table 1. Carr and Meyer (1972) and Simon *et al*.^18^ determined the petrographic mode and found a very high percentage of glass in the Apollo 14 sample 14163^16^. In addition, Matta^20^ found alkali metal elements on the Moon, which will serve as the source of the alkali activator for geopolymers. Thus, lunar soil may meet the requirements for preparing lunar cement based on geopolymer technology. If a lunar cement material utilizing lunar soil-based geopolymers for building permanent lunar bases is to be developed, then geopolymer materials must meet the following criteria: resistance to high- and low-temperature cycling, vacuum stability, minimal water requirements, good mechanical properties and sourced almost entirely from lunar resources.

The objective of this research is to verify through XRD, compressive strength measurements, and NMR tests and characterization that tektite has alkali-activated activity. The water consumption for the geopolymerization of tektites was determined using DSC and from the residual water content measured in vacuum. The cement material from the geopolymerization of tektite was tested for its durability and resistance to high- and low-temperature cycling and its vacuum stability under liquid nitrogen and vacuum conditions.

## Method

### Materials

The primary raw materials used in this study were provided by local suppliers. Lei Gong Mo, a type of black tektite found in southern China, was ground and passed through a sieve with a pore size of 74 μm. The alkaline activators used were sodium hydroxide and sodium silicate, which were produced by Xilong Chemical Company and Nanning Chunxu Chemical Company, respectively.

### Geopolymer synthesis

In this study, the 5 M NaOH solution and sodium silicate solutions (SiO_2_/Na_2_O molar ratio = 2 and solid content = 43.3%) were used as alkali activators, respectively. The mass ratios of sodium hydroxide activator/tektite powder = 0.42 and sodium silicate activator/tektite powder = 0.70. These geopolymer pastes were composed of 9.75SiO_2_-Al_2_O_3_-1.05Na_2_O-19.82 H_2_O (Na-GP) and 12.52SiO_2_-Al_2_O_3_-1.54Na_2_O-18.43 H_2_O (Na-Si-GP). First, the tektite powder was quickly mixed with the sodium hydroxide activator and the sodium silicate activator, and the preparation of fresh mixtures required intensive mixing for 2 min. Then, the samples were cast in alloy molds to yield 20 × 20 × 20 mm cubes. A thin layer of oil was sprayed into the alloy molds prior to filling to aid in the removal of the hardened paste upon curing. The alloy molds were vibrated on a vibration table for 2 min to remove any air bubbles and were sealed immediately afterwards. A total of two mixtures were prepared, and 6 samples were cast from each mixture. All of the specimens in the molds were cured under atmospheric pressure for 1 day at 60 °C. Then, the samples were unmolded and placed under vacuum (−0.096 MPa) at 60 °C until the mass of the samples did not change (12 h).

Other geopolymer pastes were synthesized by stirring the tektite powder into a solution of 5 M NaOH to give two compositions with sodium hydroxide activator/tektite powder mass ratios of 0.5 and 0.6. The mixed paste was stirred for 2 min and was then ready to be used.

### Sample characterization

The chemical compositions of the tektites were obtained by energy dispersive X-ray fluorescence (XRF) spectrometry using an Axios instrument. The sample preparation involved fusion with a 65:25:10 lithium tetraborate: lithium metaborate: lithium fluoride flux in platinum crucibles at 1050 °C for 8 minutes to produce a glass bead.

X-ray diffraction (XRD) experiments were performed using a Rigaku MiniFlex 600 instrument with Ni-filtered Cu (Kα) radiation operating at 40 kV and 15 mA with a dwell time of 3 seconds, a 2θ range of 5–70°, and a step size of 0.020°.

Scanning electron microscopy (SEM) was performed using a Hitachi S-3400N instrument at an acceleration voltage of 20 kV. The specimens were impregnated using absolute ethyl alcohol, polished with SiC paper, and then coated with gold.

In a single freeze-thaw cycle, the sample was placed in liquid nitrogen (−196 °C) for 0.5 hour and then held at 25 °C for 0.5 hour; one complete cycle took approximately 1 hour to complete. The compressive strength of the sample was determined after 30 freeze-thaw cycles. The compressive strength testing was conducted on specimens using a DNS100 universal testing machine. The displacement rate was 0.5 mm/min. To obtain the average compressive strength, three samples from each mixture were tested.

Differential scanning calorimetry (DSC) analysis was performed on powdered samples of approximately 20 mg in an alumina crucible using an STA 449 *F3* Jupiter^**®**^ (NETZSCH-Gerätebau GmbH) instrument. A heating rate of 1 °C/min between 20 °C and 130 °C with a vacuum of 2 mbar was used for the tektite powder sample. To ensure consistency between the initial states of each sample, the samples were held in the instrument at 20 °C for 20 minutes prior to commencement of the heating.

Solid-state ^29^Si MAS NMR spectra were collected at 59.62736 MHz on an Avance-300 NMR spectrometer (Bruker) using a spinning speed of 5 kHz. Solid-state ^27^Al MASNMR spectra were acquired on the same instrument at 78.20451 MHz and a spinning speed of 12.0 kHz. All data were processed using NMR-Pipe^21^. Deconvolution of the ^29^Si MAS NMR spectra was performed using Gaussian peak profiles^22^. A single Gaussian peak was used to represent each expected Q^*n*^(*m*Al) species, and these peaks were used to develop a simulation of the ^29^Si MAS NMR spectra using a least squares fitting method. Peak assignments were made with reference to the literature, and the references are provided in the main text. The minimum possible number of peaks was used to enable an accurate and meaningful interpretation of the spectra; a requirement that the intensities of adjacent peaks varied smoothly was imposed, which is consistent with the thermodynamics of a statistical distribution of Si and Al sites within an aluminosilicate network^23^,^24^.

## Results

### SEM micrographs and XRD of tektites

**Figure 1:**
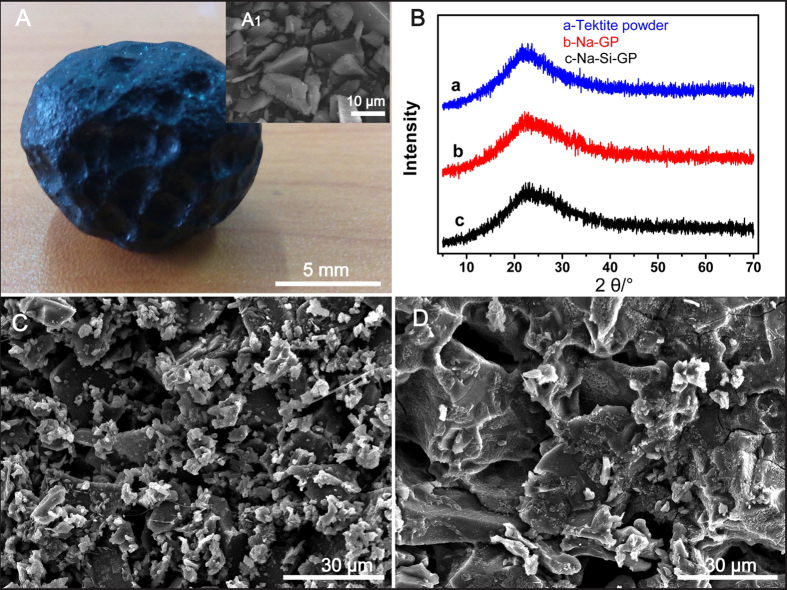
(**A**) Photo of tektite; (A_1_) an SEM image of tektite powder; (**B**) XRD patterns of (B-a) tektite powder, (B-b) Na-GP, and (B-c) Na-Si-GP; (**C**) an SEM image of Na-GP; and (**D**) an SEM image of Na-Si-GP.

The tektites’ profile picture and powder SEM image are shown in Fig. 1A and Fig. 1-A_1_. The tektites possess a dense and hard glass meteorite appearance. It has been shown through water immersion tests that tektites do not absorb water.

The XRD data collected for three types of powders are presented in Fig. 1B. The X-ray diffractograms of the tektite powder, sodium hydroxide-activated tektite and sodium silicate-activated tektite display a broad featureless hump at approximately 17–30° 2θ, which is typical of amorphous aluminosilicates. The tektite powder that was activated by NaOH and sodium silicate solutions formed a geopolymer that covered the tektite powder surface and prevented further reaction. Therefore, the tektite powders still contained a large content of unreacted glass and only a rather small amount of geopolymer. Combined with the chemical composition in Table 1, we deduce that Lei Gong Mo powder has a good alkali-activated activity because its structure is amorphous and should be readily dissolvable by alkali activators^10^,^12^,^13^,^14^. Based on the XRD phase structure and chemical composition comparison of Lei Gong Mo and lunar soil, it was determined that the structure of tektites is amorphous without a crystal phase. Lunar soil^25^,^26^ includes a glass phase and a crystal phase (plagioclase, ilmenite and augite). In addition, a previous study^27^ determined that lunar soil has a significant glass phase content, and it should exhibit geopolymerization activity. If tektite has geopolymerization activity, then we conclude that the regolith with an amorphous phase composition should also have geopolymerization activity.

**Figure 2:**
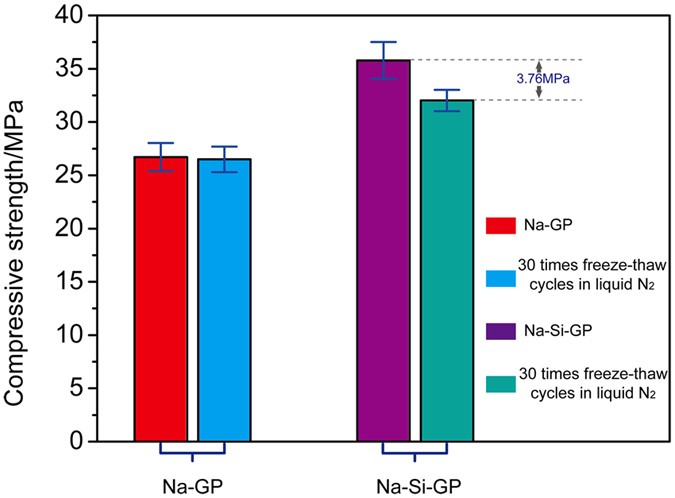
The compressive strength of the sodium hydroxide-activated and sodium silicate activated tektite powders.

Therefore, the following research is performed to demonstrate the feasibility of using geopolymer materials as lunar building materials. During alkali-activated experiments on tektite powders, it was determined that the tektite powder responded well to alkali activation. SEM images of Na-GP and Na-Si-GP are shown in Fig. 1C,D, respectively. By comparing Fig. 1C,D, it is observed that significantly more tektite powder was dissolved in Na-Si-GP than in Na-GP, and this generated more gel. Furthermore, the SEM analysis confirmed a higher amount of gel in Na-Si-GP compared to Na-GP, a higher compressive strength could be expected as obtained in Fig. 2.

### Compressive strengths of different alkali-activated tektites

**Figure 3:**
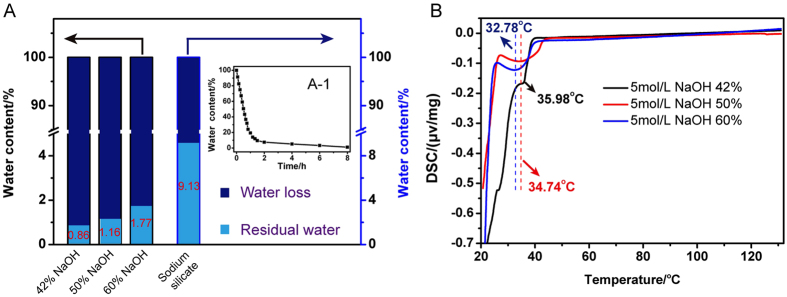
(**A**) Water content of the tektite powder activated by different amounts of sodium hydroxide and sodium silicate after heating at 120 °C under vacuum for 8 h. (A-1) The water content of the tektite powder as a function of time after adding 42% sodium hydroxide at 120 °C under vacuum conditions for 8 h. (**B**) DSC curves of the tektite powder after adding different amounts of sodium hydroxide. The curves were acquired at a 1 °C/min heating rate under vacuum conditions.

The compressive strengths of the alkali-activated tektite samples are shown in Fig. 2. The 5 M NaOH and sodium silicate solutions served as alkali-activators to form geopolymer pastes referred to as Na-GP and Na-Si-GP, respectively. The compressive strength of as-formed Na-GP was 26.71 MPa, and the compressive strength was 26.50 MPa after 30 freeze-thaw cycles in liquid nitrogen, which indicates nearly no change in the compressive strength. The compressive strength of as-formed Na-Si-GP was 35.79 MPa, and the compressive strength was 32.03 MPa after 30 freeze-thaw cycles in liquid nitrogen, resulting in a compressive strength reduction of 3.76 MPa. Combined with the SEM analysis, the NaOH activator appears to be more suitable for fabricating geopolymers than the sodium silicate solution. This result is consistent with the water contents in the Na-GP and Na-Si-GP that were measured under vacuum, as shown in Fig. 3A. In the water loss experiments, two types of geopolymer pastes solidified after curing for 8 h at 120 °C under vacuum. The residual water content in Na-Si-GP was 9.13%, which was significantly higher than that in Na-GP. Thus, the freeze-thaw durability of Na-Si-GP was worse than that of the Na-GP; thus, its compressive strength declined more after the liquid N_2_ cycles. It should be explained that the above compressive strength results are not the best data. Because of the scarcity of the raw material, few experiments have been performed.

### Residual water content of different alkali-activated and adding amount

After heating at 120 °C under vacuum for 8 h, the tektite powders treated with 42%, 50%, 60% of 5 M NaOH or 70% sodium silicate possessed a residual water content of 0.86%, 1.16%, 1.77% and 9.13%, respectively (Fig. 3A). This also confirmed the above-mentioned freeze-thaw cycle experimental result. Therefore, in this paper, sodium hydroxide is adopted as the tektite powder activator. From Fig. 3A-1, we observe that the water content of the sodium hydroxide-activated geopolymers decreased as the curing time increased, which resulted in the near complete absence of water at the end. For lunar cement materials, sodium hydroxide-activated geopolymers are the best choice. Furthermore, water consumption is only 0.86% for the entire preparation process of sodium hydroxide-activated geopolymers. Thus, based on the experimental results, we stipulate that water in the alkali-activated reaction is a medium (“porter”) and is essentially not involved in the reaction between sodium hydroxide and the tektite powder.

In Fig. 3B, the DSC curves show that the reaction of the tektite powder and sodium hydroxide is an exothermic reaction. In the experiment, the sodium hydroxide concentration was 5 mol/L. The exothermic peak position of the tektite powder after adding sodium hydroxide was reduced by 42% (35.98 °C), 50% (34.74 °C), and 60% (32.78 °C) as the sodium hydroxide amount was increased. This shows that it was easier for the reaction to proceed as the sodium hydroxide concentration increased.

### The geopolymerization mechanism of tektite

**Figure 4:**
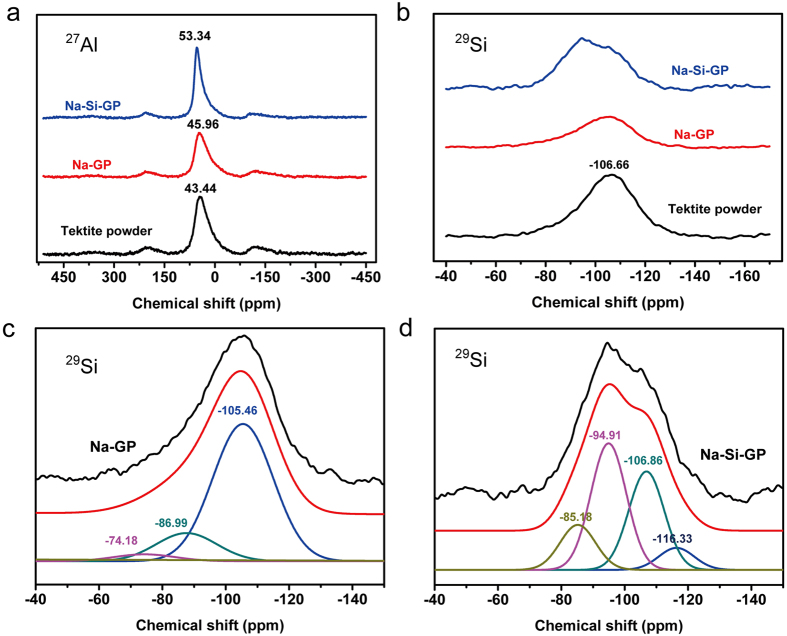
(**a**) ^27^Al and (**b**) ^29^Si MAS NMR spectra of the tektite powder, Na-GP and Na-Si-GP. For Na-GP (**c**) and Na-Si-GP (**d**), each plot is the fit of the sum of the deconvoluted peaks.

The NMR results help elucidate the geopolymerization mechanism of the aluminosilicate minerals. The NMR spectra of the tektite powder, Na-GP and Na-Si-GP are shown in Fig. 4. The ^27^Al MAS NMR spectrum for each powder is presented in Fig. 4(a). The spectra of the samples are typical of those commonly observed for aluminosilicate glasses. Furthermore, the spectra are very similar, and all of the samples display a tetrahedral Al resonance centered at approximately 50 ± 20 ppm. The tektite powder, Na-GP, and Na-Si-GP possess peaks with a chemical shift (δ_obs_) at approximately 43.44, 45.96 and 53.34 ppm, respectively^28^,^29^,^30^. The resonance attributed to Al(IV) in Na-Si-GP (δ_obs_ ~55 ppm) is within the region of the Q^4^ environments (Al bonded to four other atoms via oxygen bridges) of aluminosilicate solutions^31^,^32^, zeolites^33^ and mullite^34^. Additionally, the peak shape is sharp, and there are no other resonances. The low-molecular-weight dimer and trimer structures are excluded, which indicates that the sodium silicate-activated tektite powder forms a three dimensional network of aluminosilicate compounds.

The ^29^Si MAS NMR spectrum for each powder and the associated deconvolution for Na-GP and Na-Si-GP are presented in Fig. 4(b–d). According to the literature, when the vibrational peak of a ^29^Si spectrum is sharp and intense, the material possesses good crystallinity. Therefore, the powders are primarily in the amorphous phase and are not in the ordered crystalline phase. This is consistent with the broad amorphous hump in the XRD spectra of these samples. The smaller chemical shift of ^29^Si indicated that the degree of condensation of the SiO_4_ cells was high. The high-intensity peak that appears in the spectrum of the tektite powder at −106.66 ppm can be attributed to the Q^4^(0Al) environment^29^,^35^. The ^29^Si MAS NMR spectrum for Na-GP displays a broad resonance centered at approximately −104.5 ppm. The spectrum of each sample can be deconvoluted into resonances at −105.46 ppm and −86.99 ppm, as well as smaller contributions to the overall spectra at −74.18 ppm. The latter value is attributed to the Q^4^(0Al) and Q^4^(4Al) environments. Simultaneously, the lower coordination Si species, Q^1^(0Al), also contributes to the overall spectra. The ^29^Si MAS NMR spectrum for Na-Si-GP display a broad resonance centered at approximately −95.3 ppm. The spectrum for each sample was deconvoluted into resonances at −116.33 ppm, −106.86 ppm, −94.91 ppm and −85.18 ppm, which were attributed to Q^4^(0Al), Q^4^(2Al) and Q^4^(4Al) environments. This indicated that the coordinated Si in Na-GP and Na-Si-GP are different. There was a significant content of unreacted tektite in the geopolymer by the ^29^Si MAS NMR spectrum analysis. Through comparing the associated deconvolution for Na-GP and Na-Si-GP, we could see more Q^4^(2Al) and Q^4^(4Al) in Na-Si-GP. This demonstrated that more tektite to participate in the reaction in Na-Si-GP. The geopolymerization using the sodium silicate activator formed a three-dimension network structure more compact than that formed using the NaOH activator, as shown by the ^29^Si MAS NMR signals, and Na-Si-GP exhibited a higher compressive strength than Na-GP. However, the water consumption is greater and the resistance of Na-Si-GP to high and low temperature cycling is less than those associated with geopolymerization using the NaOH activator.

**Figure 5:**
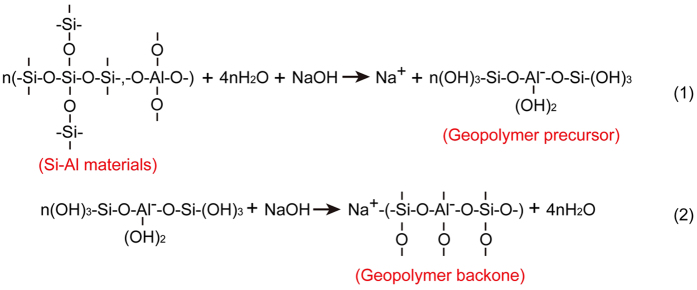
The chemical reaction mechanism between the sodium hydroxide activator and the tektite powder.

The solid-state ^27^Al and ^29^Si MAS NMR analyses of the tektite powder and the alkali-activated powders show that the mechanism of geopolymerization is identical to the one described in the literature^10^,^12^,^13^,^14^. Based on the above studies, the chemical reaction mechanism between the sodium hydroxide activator and the tektite powder is schematically presented in Fig. 5. Water acts as a catalyst during geopolymerization. Ultimately, the geopolymer molecular structure loses all of the water.

## Discussion

After studying the mechanical properties, temperature stability, and freeze-thaw durability of the tektite geopolymers under the Earth and Moon environments, it is likely that the tektite geopolymers meet most of the requirements for use in the lunar environment. Therefore, if lunar soil can be used for geopolymerization with an alkali activator, then the lunar soil geopolymer amorphous materials can be used as rock adhesives in the lunar environment to form a permanent construction^36^. Thus, the material that most likely has the potential to be composed entirely of lunar material is a geopolymer concrete. Geopolymer concrete is composed of approximately 70% to 80% aggregate and approximately 20% to 30% geopolymer binder^10^,^36^. The aggregate is fine and course regolith, and the geopolymer binder is what binds the aggregate together to make the concrete. The aggregate used in a lunar environment will be the lunar regolith, which would account for 70–80% of the materials needed for construction. If lunar soil exhibits geopolymerization activity, then more than 90% of the materials needed to produce lunar concrete could be made up of indigenous lunar materials.

**Figure 6:**
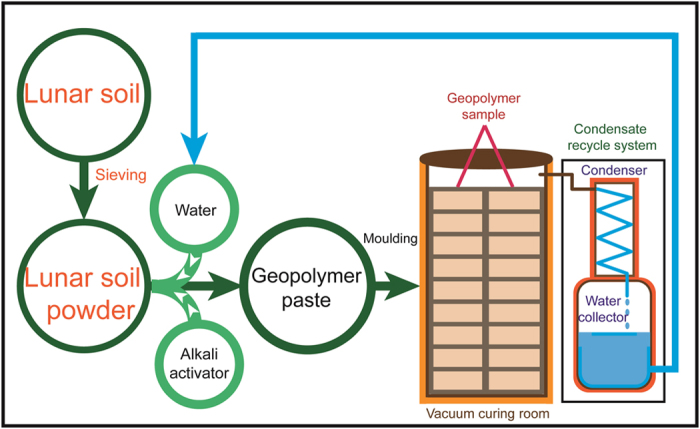
Process flow chart for the preparation of a geopolymer sample and a hypothetical water-recycling device.

A hypothetical process that can be used on the Moon to fabricate geopolymer concrete materials and a water-recycling device are shown in Fig. 6. Part I is the preparation of the geopolymer paste. First, lunar soil is sieved to obtain a higher reaction activity for the glass-phase particles. Then, appropriate amounts of water and alkali activator are added. The mixture is stirred to obtain a geopolymer paste. Part II involves the preparation of a geopolymer concrete and water recycling. The geopolymer paste is injected into a mold and is mixed with the Moon aggregates. Then, the mixture is placed in a vacuum curing room for a period of time to cure. Finally, the evaporated water is collected through the condensate recycling system and is reused. If the preparation of the geopolymer concrete for the Moon was calculated in terms of m^3^ of lunar concrete (if activated using 5 M NaOH), the final residual water in the concrete would be approximately 0.1%. The absence of a need for water to develop lunar materials is very important. Therefore, the cost and difficulty of producing construction materials on the Moon using geopolymer technology will be significantly reduced if a water recycling system is implemented. Although this study did not use lunar soil to directly synthesize geopolymers, the tektite geopolymer experiment is hypothesized to be similar. In the future, we hope to obtain lunar soil and then verify its geopolymerization and performance properties.

## Conclusions

In summary, this study determined that the tektite geopolymer meets all of the specific performance requirements for a lunar building material: resistance to high- and low-temperature cycling, vacuum stability, near-zero consumption of water, good mechanical properties and nearly exclusive use of lunar resources. Although tektite is not identical to lunar soil, lunar soil may have a similar alkali-activated performance; this is because of the glass-phase composition and lower Si/Al ratio of lunar soils, which are similar to tektites. Based on prior geopolymerization research^10^,^12^,^13^,^14^, we hypothesize that lunar soil may have a higher alkali activation activity than tektite. Therefore, currently, the development of geopolymer-based cement materials for lunar construction is a simple and feasible approach.

## Additional Information

**How to cite this article**: Wang, K.-T. *et al*. Development of near-zero water consumption cement materials via the geopolymerization of tektites and its implication for lunar construction. *Sci. Rep*. **6**, 29659; doi: 10.1038/srep29659 (2016).

## Figures and Tables

**Table 1:** Major elemental composition of tektite and its comparison with lunar samples w_B_/%.

Sample	SiO_2_	Al_2_O_3_	TiO_2_	FeO_T_	MnO	MgO	CaO	Na_2_O	K_2_O	P_2_O_5_	LOI^a^	Total
Apollo11^18^	42. 20	13. 60	7. 80	15. 30	0. 20	7. 80	11. 90	0. 47	0. 16	0. 05	0. 12	99. 90
Apollo14(14163)^18^	47.97	17. 57	1. 77	10. 41	0. 14	9. 18	11. 15	0. 70	0. 58	0. 52	—	99. 99
Apollo16^18^	45. 0	27. 30	0. 54	5. 10	0. 30	5. 7	15. 70	0. 46	0. 17	0. 11	0. 07	100. 38
Tektite [this study]	69.84	12.16	0.78	8.40	0.14	2.03	2.54	1.07	2.28	—	0.76	100.00

^a^Loss on ignition.
